# Anesthetic Management of a Patient With an Incidental Vallecular Cyst: A Case Report

**DOI:** 10.1002/ccr3.70549

**Published:** 2025-05-30

**Authors:** Jibran Ikram, Aariya Srinivasan, Ayesha Zahid, Sabry Ayad

**Affiliations:** ^1^ Outcomes Research Department Cleveland Clinic Cleveland Ohio USA; ^2^ Anesthesiology Department Cleveland Clinic Cleveland Ohio USA

**Keywords:** airway management, airway obstruction, dysphagia, vallecular cyst

## Abstract

Vallecular cysts are rare, benign lesions of the epiglottis and adjacent structures, often asymptomatic but with the potential to cause airway obstruction during anesthesia. We report the case of a 50‐year‐old female undergoing elective laparoscopic hysterectomy, during which an incidental vallecular cyst was identified during intubation. This case highlights the challenges in airway management and the need for meticulous planning to ensure patient safety. Early diagnosis and multidisciplinary management were essential for favorable outcomes.

**Summary**

- Vallecular cysts, though often asymptomatic, present significant challenges during airway management in anesthesia.
- This case underscores the critical importance of early recognition, advanced airway tools such as video laryngoscopy, and proactive strategies, including the use of airway exchangers and nasopharyngeal airways to mitigate risks.

## 1 Introduction

Vallecular cysts are mucous retention cysts of the epiglottis or vallecula, often presenting as incidental findings during imaging or airway examination. These lesions are more commonly diagnosed in children but rarely in adults, where their clinical implications may vary based on size and location [1]. Vallecular cysts may remain asymptomatic or cause nonspecific symptoms such as dysphagia, snoring, and in rare cases, airway obstruction [2]. In anesthetic practice, unexpected discoveries of airway abnormalities can significantly impact management and patient outcomes, particularly during procedures requiring endotracheal intubation. This case report describes the anesthetic management of a patient with an incidental vallecular cyst detected during a laparoscopic hysterectomy, emphasizing the importance of vigilance and tailored airway management strategies.

## 2 Case History and Examination

A 50‐year‐old female presented to the OBGYN clinic with complaints of abnormal uterine bleeding (AUB) persisting for 8–10 years. The patient had a history of multiple fibroids, polyps, anemia, and low ferritin levels managed with iron supplementation. Her past medical history included a lobectomy for idiopathic lung collapse and mild persistent asthma for which she uses a budesonide‐formoterol inhaler. She also reported occasional shortness of breath, choking, and snoring. Polysomnography ruled out obstructive sleep apnea (STOP‐BANG score: 1) and was diagnosed with primary snoring. A pulmonary function test was done in view of dyspnea and revealed a restrictive pattern with reduced forced vital capacity (FVC), though her chest X‐ray showed no abnormalities. A diffusion capacity of the lungs for Carbon Monoxide (DLCO) was done and showed reduced total lung capacity (TLC) indicating restriction and elevated RV/TLC indicating air trapping.

During the preoperative anesthesia testing (PAT) visit, her oxygen saturation was 100% on room air, blood pressure was 120/81, heart rate was 72, and respiratory rate was 16. There was no wheezing on the respiratory exam, and she was not using supplemental oxygen at home. On airway examination, Mallampati score was I, thyromental distance > 3 finger breadths, full neck range of motion, and adequate mouth opening. There were no anatomical abnormalities such as micrognathia or thick neck. Lip bite test was Grade I. No signs of airway obstruction were present on routine clinical exam. The voice was noted to be normal in quality, with no signs of vocal fatigue or altered phonation. No stridor or inspiratory noises were noted during the interview or examination.

## 3 Diagnosis and Management

The patient was scheduled for a total laparoscopic hysterectomy with bilateral salpingectomy and cystoscopy under general anesthesia. Standard American Society of Anesthesiologists (ASA) protocols were followed for monitoring vital signs.

**FIGURE 1:**
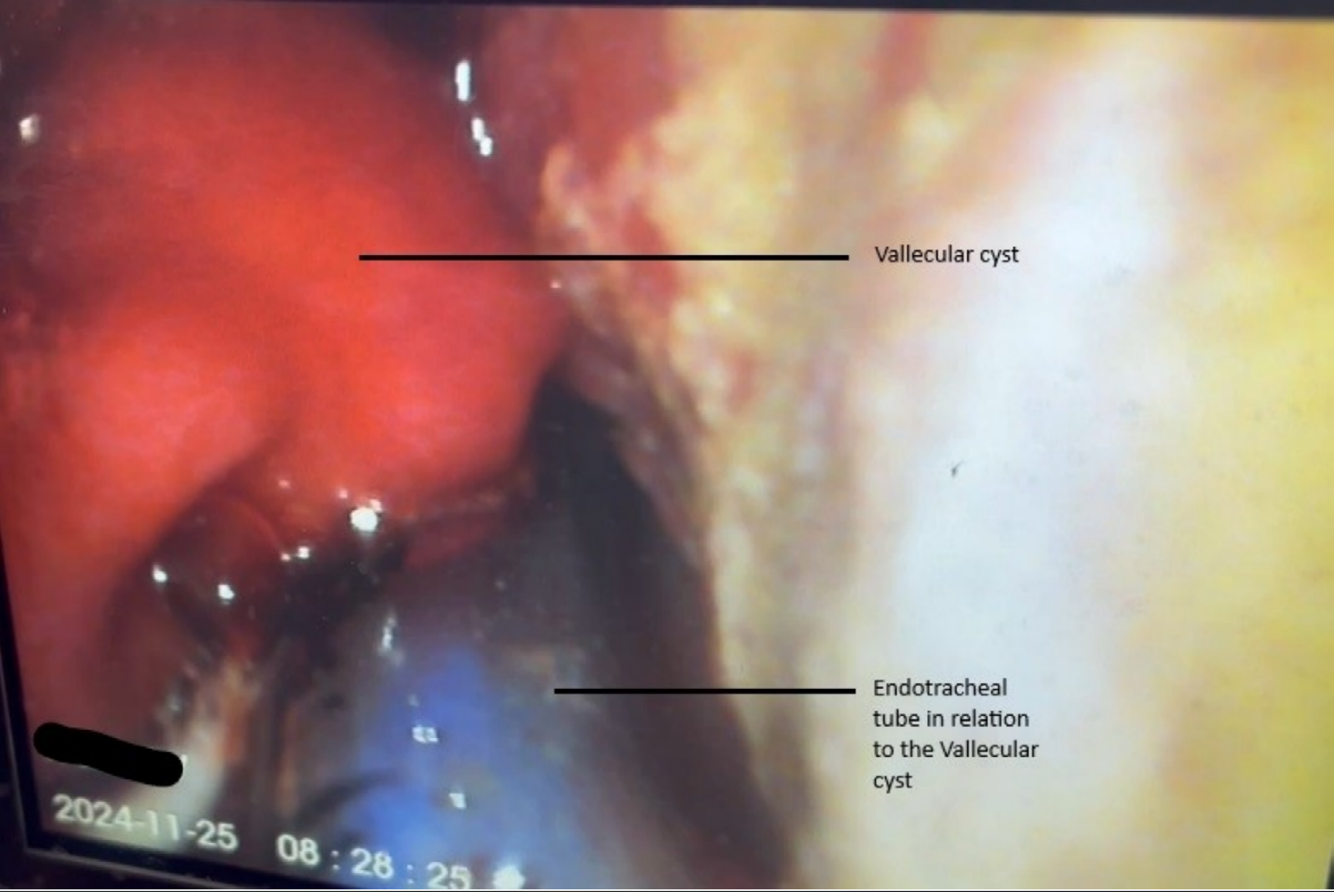
Direct laryngoscopy image showing the vallecular cyst in relation to the ET tube.

In the operating room, the patient was placed in a sniffing position and was induced with intravenous fentanyl, propofol, and rocuronium. Intubation was performed with a 7‐mm single‐lumen endotracheal tube using direct laryngoscopy. During laryngoscopy, a 2 × 2.5 cm mass above the left vocal cord was identified, later confirmed as a vallecular cyst (Figure 1). The patient was given IV 12 mg dexamethasone and hydrocortisone to reduce the edema and inflammation around the mass. The patient remained hemodynamically stable throughout the procedure.

Video laryngoscopy was used before extubation to reassess the mass. To ensure a safe emergence, a COOK airway tube exchanger was placed, and the trachea was ventilated with 100% oxygen. A nasopharyngeal airway was also inserted to maintain airway patency. The patient was awakened after a complete reversal with sugammadex and monitored closely. The patient did not have any signs of respiratory distress on extubation.

**FIGURE 2:**
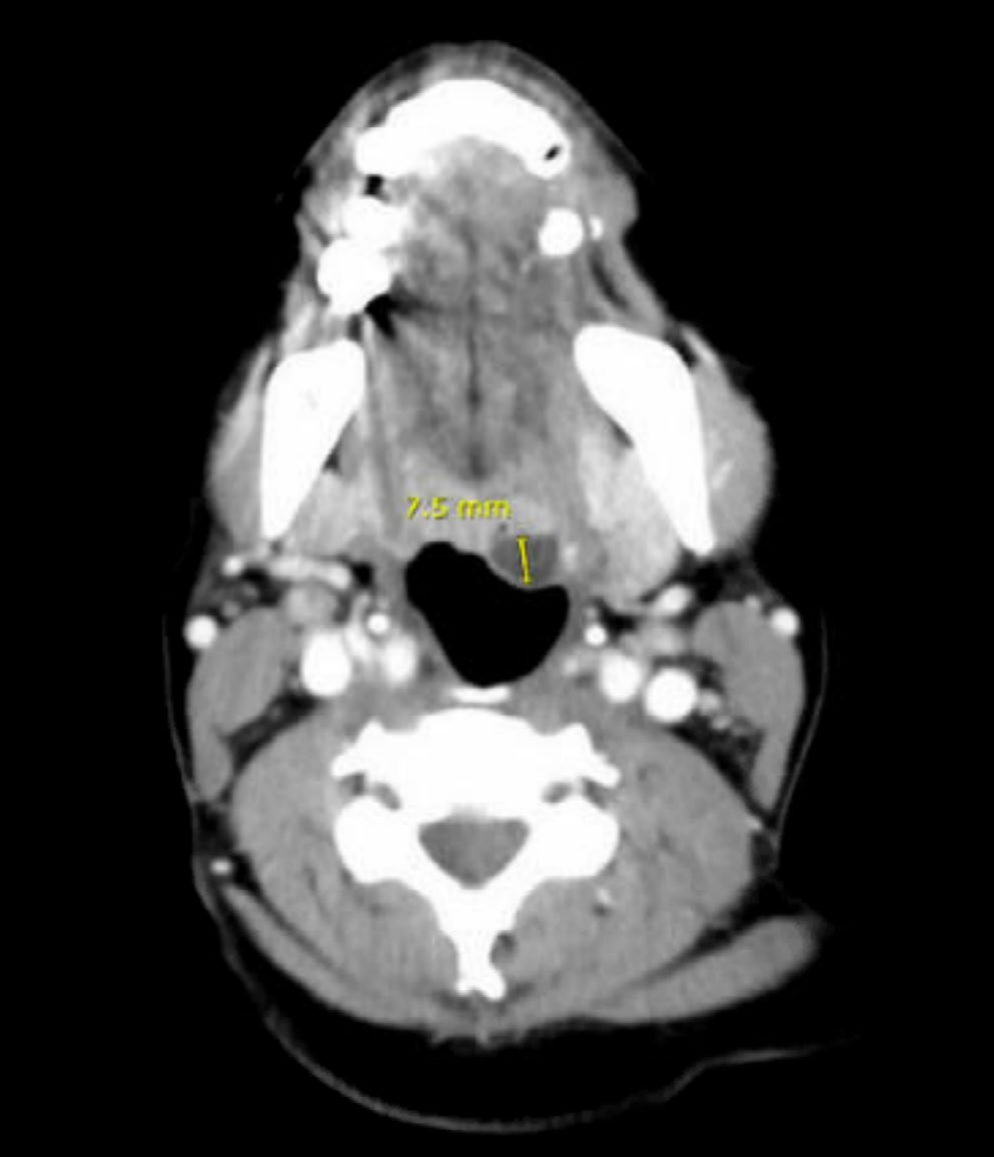
CT NECK‐soft tissues showing a 0.75 cm non‐obstructive vallecular cyst.

The patient was transferred to the recovery room on 100% oxygen and received racemic epinephrine for airway stabilization post extubation. Ear, Nose, and Throat (ENT) was consulted, and a CT scan of the neck confirmed the presence of a benign vallecular cyst measuring 0.75 cm, with no evidence of airway obstruction (Figure 2). The patient's recovery was uneventful, and she was discharged with a recommendation to follow up with an ENT specialist.

## 4 Discussion

Vallecular cysts, though rare, can pose significant challenges in airway management during anesthesia. The size of a vallecular cyst tends to increase with mucus secretion. The cyst in this patient was incidentally discovered, underscoring the importance of vigilance during airway manipulation. While these lesions are typically benign, their location near critical airway structures can lead to airway obstruction during induction, maintenance, or emergence from anesthesia [3].

Video laryngoscopy is an essential tool for visualizing airway abnormalities, aiding in both diagnosis and treatment and decision‐making [4]. This case highlights how its use facilitated a detailed examination of the vallecular cyst, ensuring a safe extubating plan. Studies have emphasized the value of advanced airway tools, particularly in unanticipated difficult airway scenarios [5].

Proactive strategies such as the use of a COOK airway tube exchanger and a nasopharyngeal airway exemplify the importance of planning for complications during extubation. In this case, although a nasopharyngeal airway was inserted to maintain upper airway patency post‐extubation, the decision to also place a COOK airway exchanger was driven by the need for a secure and immediate re‐intubation route should airway compromise occur. The presence of the vallecular cyst introduced a potential for unpredictable airway collapse or edema during emergence, making the airway exchanger a critical safety measure. While direct extubation with only a nasopharyngeal airway may be sufficient in lower‐risk scenarios, the combination of both devices provided a layered approach to airway management. This aligns with current difficult airway guidelines, which recommend the use of an airway exchange catheter when there is any concern for reintubation difficulty, even if initial intubation was straightforward [6].

Beyond real‐time airway management, anesthesiologists must also anticipate the possibility of a difficult airway when risk factors such as unexplained snoring or choking are present. Although our patient's preoperative assessment was unremarkable, subtle symptoms may warrant further airway evaluation. When vallecular cysts are suspected or discovered, options include awake fiberoptic intubation, video laryngoscopy, or maintaining spontaneous ventilation until the airway is secured [1, 5].

The importance of postoperative care, including imaging and specialist consultations, cannot be overstated. While surgical intervention may be necessary for symptomatic vallecular cysts, conservative management with monitoring is appropriate for small, benign, and non‐obstructive lesions. Collaborative management involving anesthesiologists, ENT specialists, and surgeons is pivotal for ensuring comprehensive care and favorable outcomes.

## 5 Conclusion

Vallecular cysts, while often asymptomatic, can create significant challenges in airway management during anesthesia. This case demonstrates the importance of proactive planning, advanced airway techniques such as video laryngoscopy, and collaboration among healthcare teams to ensure safe outcomes. Addressing incidental findings promptly and implementing effective perioperative strategies are critical for optimizing patient safety and care.

## Author Contributions

**Jibran Ikram:** conceptualization, writing – original draft, writing – review and editing. **Aariya Srinivasan:** conceptualization, writing – original draft, writing – review and editing. **Ayesha Zahid:** conceptualization, data curation, formal analysis, software, validation, writing – original draft, writing – review and editing. **Sabry Ayad:** conceptualization, supervision, validation, writing – review and editing.

## Consent

Written informed consent was acquired from the patient whose case details are written in the study to publish this report in accordance with the journal's patient consent policy.

## Conflicts of Interest

The authors declare no conflicts of interest.

## Data Availability

The data supporting this report's findings are available on request from the corresponding author. The data is not publicly available due to privacy or ethical restrictions.
